# Mendelian randomization study on atrial fibrillation and cardiovascular disease subtypes

**DOI:** 10.1038/s41598-021-98058-w

**Published:** 2021-09-21

**Authors:** Man Ki Kwok, Catherine Mary Schooling

**Affiliations:** 1 School of Public Health, Li Ka Shing, Faculty of Medicine, The University of Hong Kong, 1/F, Patrick Manson Building (North Wing), 7 Sassoon Road, Pok Fu Lam, Hong Kong Special Administrative Region China; 2 City University of New York Graduate School of Public Health and Health Policy, New York, USA

**Keywords:** Genetics, Cardiology

## Abstract

Atrial fibrillation (AF) has been associated with numerous diseases. However, whether AF is a cause or consequence of these diseases is uncertain. To clarify, we assessed the causal role of AF on ischemic heart disease (IHD), stroke, other cardiovascular disease (CVD) subtypes, type 2 diabetes mellitus (T2DM), and late-onset AD using bi-directional two-sample Mendelian randomization (MR) among people primarily of European descent. Genetically predicted log odds of AF was associated with any stroke (odds ratio (OR) 1.22, 95% CI 1.18 to 1.27), particularly cardioembolic stroke and possibly subdural hemorrhage, with sensitivity analyses showing similar positive findings. Genetically predicted AF was also associated with arterial thromboembolism (1.32, 1.13 to 1.53), and heart failure (1.26, 1.21 to 1.30). No association of genetically predicted AF with IHD, T2DM, cognitive function, or late-onset AD was found. Conversely, genetically predicted IHD, heart failure and possibly ischemic stroke, particularly cardioembolic stroke, were positively associated with AF. Atrial fibrillation plays a role in any stroke, arterial thromboembolism, and heart failure, corroborating current clinical guidelines on the importance of preventing these complications by effective AF management. In addition, patients with IHD, heart failure or possibly ischemic stroke might be predisposed to developing AF, with implications for management.

## Introduction

Atrial fibrillation (AF) is the most prevalent arrhythmia, affecting 0.5% of the global population (~ 33.3 million) in 2015^1^. AF is relatively common (1%) and is projected to increase in the West^2^, with sizeable hospital utilization^3^. Given AF is mostly (70%) asymptomatic, patients are often diagnosed when admitted for acute cardiac disease or stroke^4^. Patients with AF tend to develop serious complications including stroke and heart failure^5^. Clinical guidelines in North America and Europe generally recommend anticoagulants for thromboembolism prevention unless patients are at low stroke risk, and heart rate or rhythm control for cardiomyopathy prevention to lower heart failure risk^6,7^. Considering AF has been implicated in various cardiovascular diseases (CVD) and cognitive dysfunction^8^, clarifying their interrelationships will add insight into the underlying etiology and help inform clinical practice given AF is preventable and treatable.

To date, a meta-analysis of observational studies found that AF was associated with a higher risk of stroke, especially ischemic but not hemorrhagic stroke, followed by ischemic heart disease (IHD)^9^. People with type 2 diabetes mellitus (T2DM) have also been observed to have a higher risk of developing AF^10^. Observationally, AF is also linked with cognitive decline^11^ and late-onset Alzheimer’s disease (AD)^12,13^. However, observational findings on arrhythmia are known to be inherently open to unmeasured or residual confounding e.g. by socioeconomic position, and to selection bias^14^. With more definitive evidence from randomized controlled trials (RCTs), existing clinical guidelines recommend anticoagulation therapy for any stroke^7^. Specially, ischemic stroke risk was clearly reduced, but reduction in hemorrhagic stroke were less consistent perhaps depending on the type of anticoagulants^15–17^. Whether AF treatments would prevent the development of IHD remains unclear, although they may improve prognosis in people with AF and IHD^18^. Whether T2DM predisposes to AF is also uncertain^19^. Given the long latency period for developing late-onset AD, only one open-label trial of warfarin exists which showed no effect on cognitive function^20^. As such, it is important to clarify the direction of causality of these inter-relationships for more targeted and effective AF management and treatment.

To systematically examine if these diseases are causes or consequences of AF, bi-directional Mendelian randomization (MR) was used. Random allocation of genetic variants at conception is not affected by socioeconomic position and related attributes or subsequent diseases such that MR is less subject to confounding than observational studies^21^. Currently MR has mainly focused on identifying modifiable risk factors for AF, such as obesity^22^, whereas the causal role of AF in several associated diseases remains unclear. Previous MR studies have suggested ischemic stroke may have a bi-directional relationship with AF^23^ whereas T2DM unlikely affects AF^24^, with the genetic variants identified from relatively small samples (ischemic stroke: n = 10,307 cases and 19,326 controls; T2DM: n = 26,676 cases and 132,532 controls) and without consideration of ischemic stroke subtypes and associations in the other direction^25^. Another MR study did not find AF affected late-onset AD^26^, but whether the association is in the other direction and how AF relates to cognitive function has not been examined yet. Further, no MR studies have examined the relationship between AF and IHD, any stroke and its subtypes, and other CVD subtypes. To address the gap, we assessed the causal role of AF using bi-directional two-sample MR i.e., we assessed whether genetically higher risk of AF was associated with IHD, stroke (any, ischemic, hemorrhagic, and its subtypes), other CVD subtypes (arterial thromboembolism, and heart failure), T2DM, cognitive function, and late-onset AD; conversely, we assessed whether genetically higher risk of IHD, stroke, other CVD subtypes, T2DM, cognitive function, and late-onset AD were associated with AF.

## Results

### Association of genetically predicted AF with IHD, stroke, other CVD subtypes, T2DM, cognitive function, and late-onset AD

For genetic predictors of AF, we obtained 110 SNPs with the *F*-statistic = 89.3. Nearly none of the SNPs for AF and the SNPs for various outcomes (except 1 SNP for AF with cognitive function) or exposures (except 1 SNP for cardioembolic stroke and 1 SNP for heart failure with AF) were identical or highly correlated. Genetically predicted AF was unrelated to education, Townsend deprivation index, smoking, alcohol drinking, and physical activity (Table 1).

**Table 1:** Association of genetically predicted atrial fibrillation with socio-economic position (education, and Townsend deprivation index) and lifestyle (smoking, alcohol drinking, and physical activity) from the UK Biobank using Mendelian randomization (MR).

Characteristics	SNPs	*F*-statistic	Method	Mean difference	95% CI		*P*-value	IVW		MR-Egger	
								Cochran’s *Q*-statistic	*P*-value	Intercept *P*-value	I^2^
Education	110	89.3	IVW	− 0.0004	− 0.012	0.011	0.94	297.5	< 0.001		
			WM	0.002	− 0.011	0.016	0.72				
			MR-Egger	− 0.0001	− 0.023	0.023	0.99			0.97	94.7%
			MR-PRESSO	0.003	− 0.007	0.012	0.58				
Townsend	110	89.3	IVW	0.000	− 0.009	0.009	0.98	175.8	0.0001		
Deprivation index			WM	− 0.003	− 0.016	0.010	0.65				
			MR-Egger	− 0.005	− 0.023	0.013	0.61			0.55	94.6%
			MR-PRESSO	0.001	− 0.008	0.010	0.82				

				Odds ratio	95% CI		*P*-value	IVW		MR-Egger	
								Cochran’s *Q*-statistic	*P*-value	Intercept *P*-value	I^2^
Alcohol drinking	110	89.3	IVW	0.998	0.996	1.000	0.04	147.6	0.01		
			WM	0.999	0.995	1.002	0.35				
			MR-Egger	1.000	0.996	1.004	0.99			0.25	94.7%
			MR-PRESSO	0.998	0.996	1.000	0.04				
Smoking	110	89.3	IVW	1.001	0.998	1.004	0.38	173.3	0.0001		
			WM	1.001	0.998	1.005	0.42				
			MR-Egger	1.005	0.999	1.010	0.09			0.15	94.7%
			MR-PRESSO	1.001	0.998	1.004	0.39				
Physical activity	110	89.3	IVW	1.004	0.983	1.026	0.71	174.0	0.0001		
			WM	0.996	0.968	1.025	0.78				
			MR-Egger	1.003	0.961	1.048	0.89			0.96	94.7%
			MR-PRESSO	1.002	0.984	1.022	0.80				

CI, confidence interval; IVW, inverse variance weighting; MR, Mendelian randomization, SNP, single nucleotide polymorphism; WM, weighted median.

^a^MR-PRESSO estimate was obtained by excluding 4 outliers (*rs12245149, rs2040862, rs35005436, rs3820888*) for education, 1 outlier (*rs6596717*) for Townsend deprivation index, 3 outliers (*rs11191116, rs1458038, rs6596717*) for physical activity.

**Figure 1:**
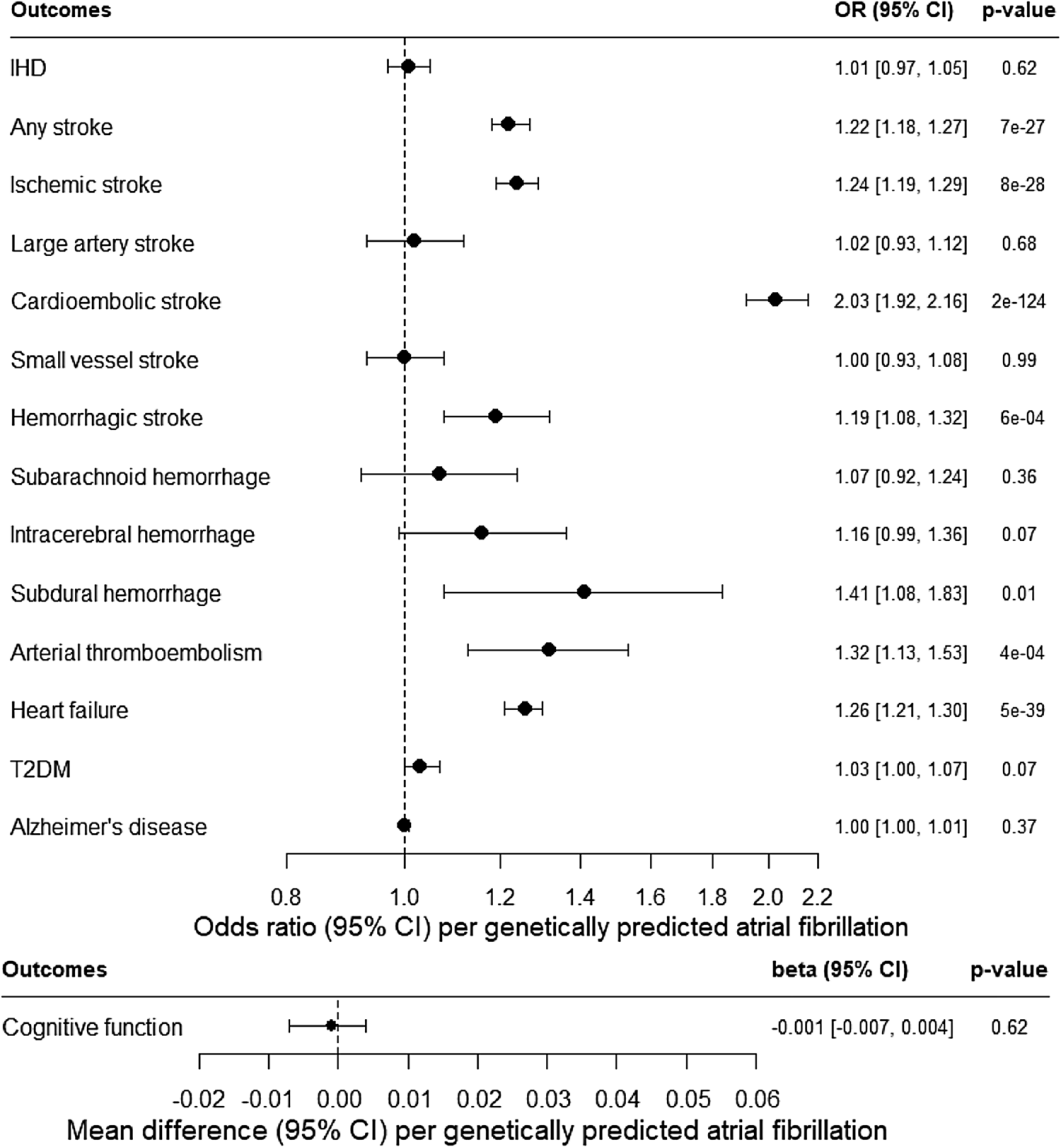
Association of genetically predicted atrial fibrillation with ischemic heart disease (IHD), stroke, arterial thromboembolism, heart failure, type 2 diabetes (T2DM), cognitive function, and late-onset Alzheimer’s disease (AD) based on estimates from inverse variance weighting (IVW) using Mendelian randomization (MR).

Figure 1 (and Appendix Table 1) shows genetically predicted AF was not associated with IHD, but was consistently positively associated with any stroke, and major stroke sub-types: ischemic stroke and possibly hemorrhagic stroke. In particular, genetically predicted AF was positively associated with cardioembolic stroke at Bonferroni-corrected significance and subdural hemorrhage at nominal significance, and perhaps intracerebral hemorrhage (although its confidence interval included the null value). No association of genetically predicted AF with large artery stroke, small vessel stroke or subarachnoid hemorrhage was found. Genetically predicted AF was also positively associated with arterial thromboembolism and heart failure. Sensitivity analyses using a weighted median, MR-Egger and/or MR-PRESSO showing similar positive findings. Genetically predicted AF was not associated with T2DM, cognitive function, or late-onset AD.

### Association of genetically predicted IHD, stroke, other CVD subtypes, T2DM, cognitive function, and late-onset AD with AF

**Figure 2:**
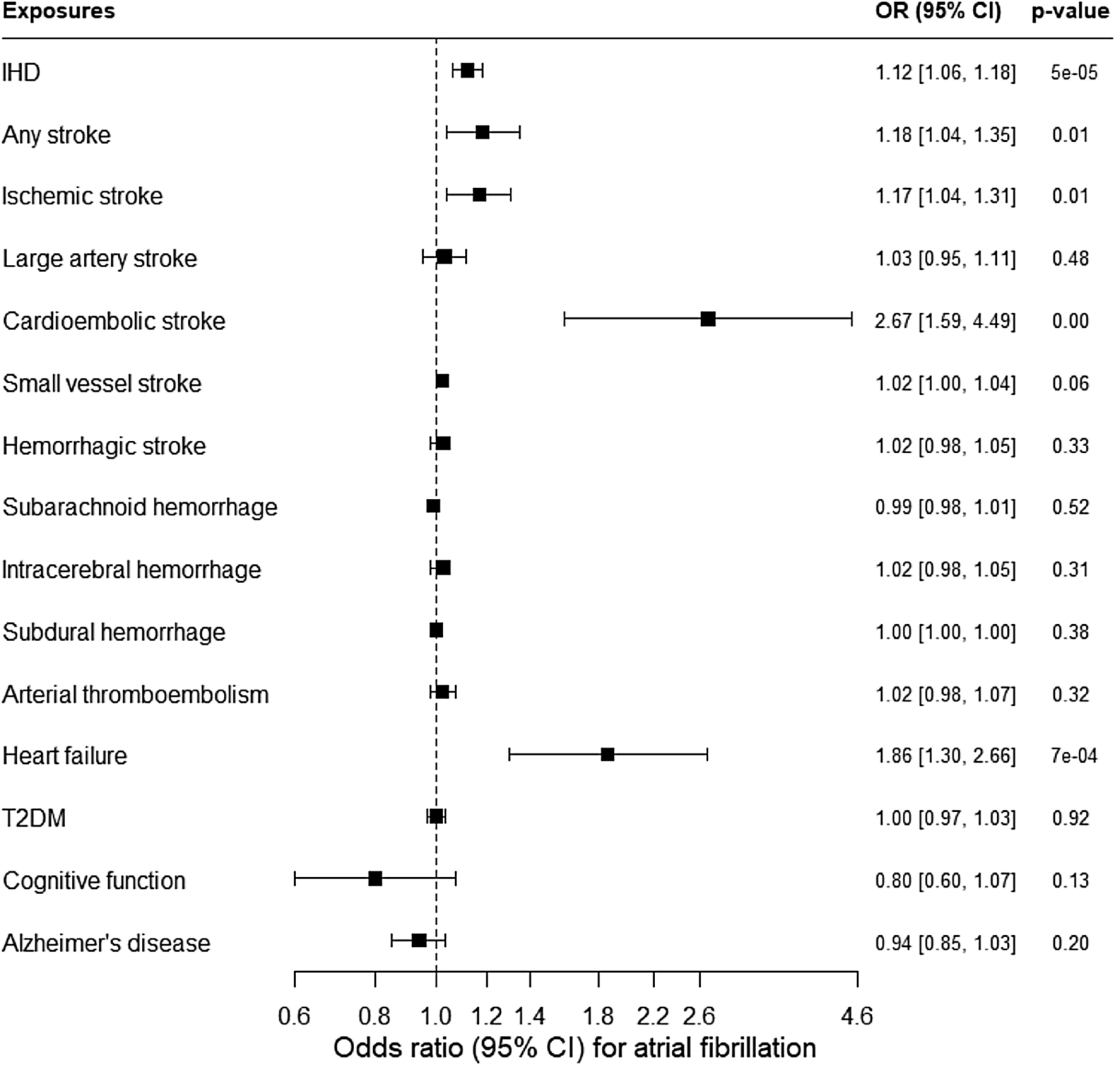
Association of genetically predicted ischemic heart disease (IHD), stroke, arterial thromboembolism, heart failure, type 2 diabetes (T2DM), cognitive function, and late-onset Alzheimer’s disease (AD) with atrial fibrillation based on estimates from inverse variance weighting (IVW) using Mendelian randomization (MR).

Figure 2 (and Appendix Table 2) shows genetically predicted IHD was positively associated with AF. Genetically predicted stroke (possibly any and ischemic, but not hemorrhagic) was also positively associated with AF at nominal significance. In particular, genetically predicted cardioembolic stroke and heart failure were positively associated with AF at Bonferroni-corrected significance, and perhaps small vessel stroke (although its confidence interval included the null value). No association of genetically predicted large artery stroke, subarachnoid hemorrhage, intracerebral hemorrhage or subdural hemorrhage with AF was found. Sensitivity analyses using a weighted median, MR-Egger and/or MR-PRESSO showing similar positive findings. No association of genetically predicted arterial thromboembolism, T2DM, cognitive function, or late-onset AD with AF was found. After excluding the overlapping SNP, similar patterns of association for AF with cognitive function and for cardioembolic stroke with AF were found (Appendix Table 3). Scatter plots showing associations for AF with these cardiovascular diseases and vice versa are shown in Appendix Figures 1 and 2.

Power calculations showed that this study had 80% power to detect OR ranging from 1.10 to 2.77 for the associations of AF with various diseases, and OR ranging from 1.10 to 1.71 for the associations of various diseases with AF (Appendix Table 4).

## Discussion

This first two-sample MR study assessing the bi-directional relationships between AF and CVD, T2DM, cognitive function, and late-onset AD suggests that AF may have a bi-directional relationship with any or ischemic stroke, particularly cardioembolic stroke. Further, AF may be a consequence of IHD that causes possibly hemorrhagic stroke, particularly subdural hemorrhage, arterial thromboembolism, and heart failure, but not T2DM, cognitive function, or late-onset AD. Our MR findings corroborate current clinical guidelines on the importance of preventing arterial thromboembolism, any stroke, and heart failure for effective AF management^6,7^, given these diseases are likely complications of AF. These findings also suggest people with IHD, heart failure and possibly ischemic stroke may be more susceptible to develop AF, given these diseases are likely the causes of AF, with implications for management of AF.

Overall, our MR findings support the causal role of AF in stroke and its subtypes. Consistent with the MR of the bi-directional association between ischemic stroke and AF^23^, we found ischemic stroke, particularly cardioembolic stroke (and possibly small vessel stroke), predisposes to AF or vice versa. There was no association of hemorrhagic stroke and its subtypes with AF, which might be underpowered due to few cases in Western populations and future replication is warranted when a larger GWAS of hemorrhagic stroke and its subtypes becomes available. We add that AF may cause any stroke and its major subtypes, with both ischemic stroke (especially cardioembolic stroke) and possibly hemorrhagic stroke (subdural hemorrhage, and perhaps intracerebral hemorrhage) being potential consequences. Further, this study provides evidence that AF predisposes to higher risk of arterial thromboembolism, and heart failure, corresponding to current clinical guidelines on the prevention of AF complications^6,7^. In contrast, this study clarifies that AF unlikely predisposes to IHD, but actually IHD may increase AF, coherent with the RCT showing better prognosis (fewer strokes) in people with AF and IHD when receiving anticoagulants^18^. Consistent with the previous MR study on the association of T2DM with AF^24^, we found no association between AF and T2DM (bi-directionally) using genetic variants of T2DM identified from a large sample. In addition, our MR suggests AF unlikely affects cognitive function or, consistent with another MR study^26^, late-onset AD, substantiating the null effect in an open-label trial of warfarin on cognitive function^20^. Our findings are less consistent with observational studies reporting AF associated with any or ischemic (not hemorrhagic) stroke, IHD^9^, cognitive decline^11^, and late-onset AD^12,13^, whereas people with T2DM develop more AF^10^. However, observational studies in patients are difficult to interpret because of selection bias.

Taken together, these findings suggest a potentially causal link from IHD and possibly ischemic stroke (especially cardioembolic stroke, and perhaps small vessel stroke) to AF, as well as AF to ischemic (especially cardioembolic stroke) and possibly hemorrhagic stroke (subdural hemorrhage, and perhaps intracerebral hemorrhage), arterial thromboembolism, and heart failure, but not T2DM or late-onset AD. AF may be the consequence of damage to the heart via atrial ischemia/infarction from IHD^27,28^, as well as dysregulation of heart rate via impaired autonomic nervous system from stroke^29,30^. AF may contribute to thrombosis and hence arterial thromboembolism because more turbulent blood flow resulting from an irregular heart beat could damage endothelial structure/function and cause blood stasis, hence promoting coagulation^31^. In particular, AF likely facilitates the coagulation cascade rather than platelet aggregation, considering the failure of aspirin to reduce stroke risk in AF^32^ or coagulation biomarkers among patients with AF^33^. Further, AF was not only related to ischemic stroke (particularly cardioembolic stroke), but also to possibly hemorrhagic stroke (subdural hemorrhage, and perhaps intracerebral hemorrhage) in this MR study. AF is known to increase cardioembolic stroke, considering abnormal atrial contraction may result in blood stasis within the left atrium and hence embolism, which could subsequently translocate to the brain^34^. For stroke prevention, anticoagulant prescription is recommended among AF patients, but control within the therapeutic range is essential to reduce thrombotic risk when under-dose or bleeding risk when overdose^35^. Vitamin K antagonists (e.g. warfarin) have been shown to promote hemorrhagic stroke, rather than other anticoagulants (e.g. direct factor Xa inhibitor)^36,37^. As such, AF may also increase risk of subdural and possibly intracerebral hemorrhage due to anticoagulant elicited bleeding risk. In addition, AF was unrelated to IHD or T2DM in our study, indicating atherosclerosis or hyperglycemia may be unlikely to be involved. The specific association of AF with ischemic stroke but not IHD lends credence to the relevance of coagulation rather than atherosclerosis, coherent with few coagulation factors being linked with IHD in a two-sample MR study^38^. Despite the lack of association of AF with IHD, AF was associated with heart failure and vice versa. AF may generate cardiomyopathy^39^ and hence increase the risk of developing heart failure^40^. Conversely, heart failure may induce atrial remodeling and hence predispose to AF^41^. Considering the pathophysiology of AF is complex and multifactorial, this first MR study adds etiological insights for further examination so as to dissect out causes and consequences of AF.

Some limitations have to be considered. First, we obtained independent SNPs reaching genome-wide significance that predict AF from the largest GWAS, with *F*-statistic greater than 10 and sufficient power to detect small effect sizes on various diseases (OR ranging from 1.10 to 2.77). Conversely, unlike SNPs that predicted IHD, any and ischemic stroke, T2DM, cognitive function, and late-onset AD, fewer SNPs that predicted rarer outcomes including hemorrhagic stroke, arterial thromboembolism and heart failure were available based on the relatively smaller number of participants having these conditions from the UK Biobank whose participants were generally healthier than the underlying population^42^. However, our *F*-statistic for all these SNPs > 10 indicates low possibility of weak instrument bias. Second, considering the pathophysiology between AF and CVD, T2DM and late-onset AD remains to be elucidated, we did not exclude some possible pleiotropic effects because they may arguably be potential mediators (e.g. BMI) such that removing these SNPs might not produce robust causal estimates. Nonetheless, the null findings remain similar before and after considering any statistical evidence of pleiotropic outliers based on the weighted median, MR-Egger and MR-PRESSO^43^. Third, we used several sensitivity analyses to validate the IVW results. The positive association of any and ischemic stroke with AF using IVW was in the opposite direction using MR-Egger. MR-Egger is sensitive to outliers that may reverse the sign of the estimates especially the limited number of SNPs^44^, whereas the weighted median and MR-PRESSO methods, which take outliers into account, obtained results that were more consistent with the IVW approach. Fourth, selection bias might bias estimates concerning late onset conditions that share etiology with common conditions that cause death prior to recruitment^45^, possibly attenuating estimates for late-onset conditions, so that estimates may be conservative. Fifth, sample overlap in two-sample MR might bias in the direction of confounding, particularly for weak instruments^46^. However, most instruments had acceptable *F*-statistics ranging from 22.1 to 163.6. We also used the largest available non-overlapping GWAS for IHD. Sixth, the AF GWAS does not account for medication usage. AF patients with higher risk of developing stroke may be prescribed anticoagulants, however medication use does not confound genetic associations although not adjusting for medication use might impair precision. Seventh, although this study showed no association of AF with cognitive function or late-onset AD, we cannot rule out that AF may affect cognitive decline, which warrants further investigation. Finally, the applicability of our findings based on largely on people of European descent to other populations including Chinese needs further investigation, considering the relevance of a causal factor may vary by setting.

From a clinical perspective, our findings suggest AF may predispose to both ischemic (particularly cardioembolic stroke) and possibly hemorrhagic stroke (subdural hemorrhage), arterial thromboembolism, and heart failure. These findings emphasize the importance of addressing the current underutilization of effective treatment for AF (anticoagulants)^47^, which could help prevent serious cardiovascular complications. Further, IHD, heart failure and possibly ischemic stroke (particularly cardioembolic stroke) could predispose to AF, with corresponding implications for management. The null finding of AF on late-onset AD casts doubt on the relevance of AF treatment in late-onset AD. Together with the potential of technological innovation in early detection of often asymptomatic AF^48,49^, better understanding of AF etiology will contribute to tackling the growing societal burden of AF in a more targeted and effective manner.

## Methods

### Data source

#### Association of genetically predicted AF with IHD, stroke, other CVD subtypes, T2DM, cognitive function, and late-onset AD

We obtained genetic predictors of AF from the largest genome-wide association study (GWAS) by Nielsen et al. (2018) (n = 60,620 cases and 970,216 controls) almost entirely in people of European descent (98.6%) adjusted for age, sex, study-specific covariates, and if available, principal components^50^. AF was identified mainly based on clinical diagnosis codes in medical records (ICD-9 and ICD-10 codes) supplemented with 12-lead electrocardiogram at the examination. Genetic associations with IHD were from CARDIoGRAMplusC4D 1000 Genomes-based GWAS^51^, with stroke from MEGASTROKE (any, ischemic, large artery, cardioembolic, small vessel)^52^ and the UK Biobank SAIGE study (hemorrhagic, subarachnoid, intracerebral, subdural)^53^, with other CVD subtypes from the UK Biobank SAIGE study (arterial thromboembolism)^53^ and HERMES GWAS (heart failure)^54^, with T2DM from DIAMANTE^55^, with cognitive function from Davies et al. (2018)^56^, and with late-onset AD from Jansen et al. (2019)^57^.

#### Association of genetically predicted IHD, stroke, other CVD subtypes, T2DM, cognitive function, and late-onset AD with AF

Genetically predicted IHD were obtained from CARDIoGRAMplusC4D 1000 Genomes-based GWAS (n = 60,801 cases and 123,504 controls) among people primarily of European descent (77%), adjusted for genomic control^51^. Genetic predictors of any and ischemic stroke were from MEGASTROKE (any: n = 40,585 cases, ischemic: n = 34,217 cases, large artery: n = 4373 cases, cardioembolic: n = 7193 cases, small vessel: n = 5386 cases, and 406,111 controls) (mean age 67.4 years, 41.7% women from the full trans-ethnic studies including Europeans), adjusted for age, sex and study-specific covariates and corrected for genomic control^52^. Genetic predictors of hemorrhagic stroke were from the UK Biobank SAIGE study (hemorrhagic: n = 1796 cases, subarachnoid: n = 812 cases, intracerebral: n = 700 cases, subdural: n = 259 cases, and 399,017 controls) among people of British white descent, adjusted for birth year, sex, and four principal components^53^. The UK Biobank recruited 503,317 adults (94% European ancestry) intended to be aged 40 to 69 years between 2006 and 2010^42^. Genetic predictors of arterial thromboembolism were from the UK Biobank SAIGE study (n = 921 cases and 400,595 controls)^53^. Genetic predictors of heart failure were from Heart Failure Molecular Epidemiology for Therapeutic Targets (HERMES) GWAS (n = 47,309 cases and 930,014 controls) (mean age: cases 71.4 years and controls 52.4 years), adjusted for age and sex, and if available, principal components and genomic control^54^. Genetic predictors of T2DM among people of European descent only were obtained from DIAbetes Meta-ANalysis of Trans-Ethnic association studies (DIAMANTE) (n = 74,124 cases and 824,006 controls) (mean age: cases 55.2 years and controls 52.7 years; proportion of women: cases 49.6% and controls 48.0%), adjusted for study-specific covariates and principal components, and corrected for genomic control^55^. Genetic predictors of cognitive function were from Davies et al. (2018)^56^ (n = 300,486) among people without stroke or dementia of European descent (age range 16–102 years), adjusted for age, sex, population stratification, and cohort-specific covariates. Genetic predictors of late-onset AD were from Jansen et al. (2019) (n = 79,145 late-onset AD cases, 47,793 proxy cases without late-onset AD but with family history of late-onset AD and 328,320 proxy controls without late-onset AD or family history of AD) among people of European descent (mean age of onset for late-onset AD and proxy cases = 74.1 years and mean age of last contact for proxy controls = 68.5 years), adjusted for sex and/or age or study-specific covariates^57^. Genetic associations with AF were from Nielsen et al. (2018)^50^.

### Statistical analysis

MR has three fundamental assumptions: (a) relevance requires the genetic variants are associated with the exposure; (b) independence refers to the genetic variants being unrelated to confounders of the exposure-outcome association; and (c) exclusion-restriction means the genetic variants affect the outcome only via the exposure^58^. We selected genetic predictors as single nucleotide polymorphisms (SNPs) genome-wide significantly (*P* < 5 × 10^−8^) and independently (r^2^ < 0.001) associated with each exposure; except a less stringent level of significance (*P* < 5 × 10^−6^) for any stroke and its subtypes, and arterial thromboembolism due to the lack of genome-wide significant SNPs. Independent variants (r^2^ < 0.001) were selected using the “clump_data” function of the MR-Base R package (http://www.mrbase.org/). Non-bialleleic or indel genetic variants or those without a rs number were excluded. Proxy SNPs (r^2^ ≥ 0.8) in Europeans obtained from LDLink^59^ were used for any SNP unavailable for the outcome. Palindromic SNPs coded A/T or C/G were aligned on effect allele frequency, for minor allele frequency ≤ 42%. Any overlapping between SNPs for AF and SNPs for various outcomes (and vice versa) were checked; if the SNPs were identical or highly correlated (linkage disequilibrium r^2^ ≥ 0.8), as sensitivity analyses, we repeated the analyses after excluding these SNPs. We computed the *F*-statistic for SNPs on the exposure to assess instrument strength (i.e., relevance). We identified any association of genetic predictors of atrial fibrillation with possible confounders (education, Townsend deprivation index, smoking, alcohol drinking, and physical activity) in the UK Biobank to assess whether the genetic predictors were unconfounded (i.e., independence).

For MR estimation, as the main analysis, inverse variance weighting (IVW) with multiplicative random-effects, which assumes balanced horizontal pleiotropy (i.e., random positive or negative pleiotropy exist with zero average pleiotropic effect), was used^60^. Compared with IVW with additive random-effects, it is less subject to weak instrument bias (by downweighing estimates from SNPs with weaker SNP-exposure associations) and is relatively robust to outliers (by assigning weights to estimates based on standard error of SNP–outcome associations)^61^. We combined SNP-specific Wald estimates (which were calculated as the genetic association with the outcome divided by genetic association with the exposure) using inverse variance weighting (IVW) with multiplicative random-effects, from which odds ratio (OR) or beta coefficients (mean differences) with 95% confidence intervals (CIs) and Cochran’s *Q*-statistic and *P*-value for heterogeneity were presented^61^. As sensitivity analyses to assess horizontal pleiotropy (i.e., exclusion-restriction), we used three complementary methods: (1) A weighted median which requires at least 50% of the information from valid SNPs. (2) MR-Egger which allows all SNPs to be invalid provided that the InSIDE (Instrument Strength Independent of Direct Effect) assumption holds, from which an intercept with *P* < 0.05 indicates the presence of pleiotropy and a higher I^2^ value indicates the ‘no measurement error’ assumption holds^62^. (3) Mendelian Randomization Pleiotropy RESidual Sum and Outlier (MR-PRESSO) identifies potentially pleiotropic outliers and provides estimates after excluding these outlier SNPs^43^. Power calculations were performed to estimate the minimally detectable effect size for MR of AF with a power of 80% and a significance level of 0.05^63^.

For assessing the bi-directional associations of AF with cardiovascular outcomes, to adjust for multiple comparisons, a Bonferroni-corrected significance level of 0.0031 (0.05/16) was considered to account for testing inter-relationships between AF and eight traits (i.e., IHD, ischemic stroke, hemorrhagic stroke, arterial thromboembolism, heart failure, T2DM, cognitive function, and late-onset AD).

We conducted statistical analyses using R version 4.0.1 (R Foundation for Statistical Computing) with the MendelianRandomization and MRPRESSO R packages unless specified.

### Ethics approval

Each study has been specifically approved by the Ethical Committees of the original studies and all the participants provided a written informed consent. This analysis of publicly available summary data does not require ethical approval. Procedures were performed in accordance with the Declaration of Helsinki.

## Supplementary Information

Supplementary Information.

## Data Availability

Data are available in public, open access repositories with the repository names and/or URL listed in Acknowledgement.

## Abbreviations

AD: Alzheimer's disease
AF: Atrial fibrillation
CVD: Cardiovascular disease
GWAS: Genome-wide association study
IVW: Inverse variance weighting
IHD: Ischemic heart disease
LD: Linkage disequilibrium
MR: Mendelian randomization
SNP: Single nucleotide polymorphism
T2DM: Type 2 diabetes mellitus
WM: Weighted median
