# Supplementary material for: Mendelian randomization study on atrial fibrillation and cardiovascular disease subtypes
Source: Sci Rep. 2021 Sep 21;11:18682. doi: 10.1038/s41598-021-98058-w (PMC8455674; doi:10.1038/s41598-021-98058-w)
Supplement: Supplementary file 1 — Supplementary Information. [file 41598_2021_98058_MOESM1_ESM.docx]

**Online supplementary information**

**Mendelian randomization study on atrial fibrillation and cardiovascular disease subtypes**

Man Ki Kwok, PhD,^1^ Catherine Mary Schooling,* PhD^1,2^

**Author affiliations**:

^1^ School of Public Health, Li Ka Shing Faculty of Medicine, The University of Hong Kong, Hong Kong Special Administrative Region, China

^2^ City University of New York Graduate School of Public Health and Health Policy, New York, United States

***Correspondence:** [cms1@hku.hk](mailto:cms1@hku.hk)

Appendix Table 1. Association of genetically predicted atrial fibrillation with ischemic heart disease (IHD), stroke, arterial thromboembolism, heart failure, type 2 diabetes (T2DM), cognitive function, and late-onset Alzheimer’s disease (AD) using Mendelian randomization (MR)

| Outcomes | Sources | SNPs | *F*-statistic | Method | Odds | 95% CI |  | *P*-value | IVW | |  | MR-Egger | |
| --- | --- | --- | --- | --- | --- | --- | --- | --- | --- | --- | --- | --- | --- |
|  |  |  |  |  | ratio |  |  |  | Cochran’s  *Q*-statistic | *P*-value |  | Intercept  *P*-value | I^2^ |
| IHD | 1000 Genomes | 108 | 90.0 | IVW | 1.01 | 0.97 | 1.05 | 0.62 | 204.3 | <0.001 |  |  |  |
|  |  |  |  | WM | 1.00 | 0.95 | 1.05 | 0.98 |  |  |  |  |  |
|  |  |  |  | MR-Egger | 0.95 | 0.88 | 1.02 | 0.13 |  |  |  | 0.04 | 95.1% |
|  |  |  |  | MR-PRESSO^a^ | 1.00 | 0.97 | 1.04 | 0.80 |  |  |  |  |  |
| Any stroke | MEGASTROKE | 109 | 89.7 | IVW | 1.22 | 1.18 | 1.27 | <0.001 | 195.2 | <0.001 |  |  |  |
|  |  |  |  | WM | 1.24 | 1.18 | 1.30 | <0.001 |  |  |  |  |  |
|  |  |  |  | MR-Egger | 1.20 | 1.12 | 1.29 | <0.001 |  |  |  | 0.56 | 95.0% |
|  |  |  |  | MR-PRESSO^a^ | 1.22 | 1.17 | 1.26 | <0.001 |  |  |  |  |  |
| Ischemic stroke | MEGASTROKE | 109 | 89.7 | IVW | 1.24 | 1.19 | 1.29 | <0.001 | 180.6 | <0.001 |  |  |  |
|  |  |  |  | WM | 1.27 | 1.20 | 1.34 | <0.001 |  |  |  |  |  |
|  |  |  |  | MR-Egger | 1.23 | 1.14 | 1.32 | <0.001 |  |  |  | 0.84 | 94.9% |
|  |  |  |  | MR-PRESSO^a^ | 1.23 | 1.19 | 1.27 | <0.001 |  |  |  |  |  |
| Large artery | MEGASTROKE | 110 | 89.7 | IVW | 1.02 | 0.93 | 1.12 | 0.68 | 164.8 | <0.001 |  |  |  |
| stroke |  |  |  | WM | 1.07 | 0.93 | 1.23 | 0.37 |  |  |  |  |  |
|  |  |  |  | MR-Egger | 0.98 | 0.82 | 1.18 | 0.85 |  |  |  | 0.65 | 94.9% |
|  |  |  |  | MR-PRESSO^a^ | 1.07 | 0.98 | 1.16 | 0.14 |  |  |  |  |  |
| Cardioembolic | MEGASTROKE | 110 | 89.7 | IVW | 2.03 | 1.92 | 2.16 | <0.001 | 117.7 | 0.27 |  |  |  |
| stroke |  |  |  | WM | 2.15 | 1.95 | 2.36 | <0.001 |  |  |  |  |  |
|  |  |  |  | MR-Egger | 2.31 | 2.06 | 2.58 | <0.001 |  |  |  | 0.01 | 95.0% |
|  |  |  |  | MR-PRESSO^a^ | 2.03 | 1.92 | 2.16 | <0.001 |  |  |  |  |  |
| Small vessel | MEGASTROKE | 110 | 89.7 | IVW | 1.00 | 0.93 | 1.08 | 0.99 | 138.3 | 0.03 |  |  |  |
| stroke |  |  |  | WM | 0.98 | 0.88 | 1.10 | 0.76 |  |  |  |  |  |
|  |  |  |  | MR-Egger | 0.92 | 0.79 | 1.07 | 0.29 |  |  |  | 0.22 | 94.9% |
|  |  |  |  | MR-PRESSO^a^ | 0.99 | 0.92 | 1.07 | 0.84 |  |  |  |  |  |

Appendix Table 1. (Continued)

| Outcomes | Sources | SNPs | *F*-statistic | Method | Odds | 95% CI |  | *P*-value | IVW | |  | MR-Egger | | |  |
| --- | --- | --- | --- | --- | --- | --- | --- | --- | --- | --- | --- | --- | --- | --- | --- |
|  |  |  |  |  | ratio |  |  |  | Cochran’s  *Q*-statistic | *P*-value |  | Intercept  *P*-value | I^2^ | |  |
| Hemorrhagic | UK Biobank | 110 | 89.3 | IVW | 1.19 | 1.08 | 1.32 | 0.001 | 89.6 | 0.91 |  |  | |  | |
| stroke |  |  |  | WM | 1.26 | 1.06 | 1.48 | 0.01 |  |  |  |  | |  | |
|  |  |  |  | MR-Egger | 1.19 | 0.97 | 1.46 | 0.10 |  |  |  | 0.97 | | 94.7% | |
|  |  |  |  | MR-PRESSO | 1.19 | 1.09 | 1.31 | <0.001 |  |  |  |  | |  | |
| Subarachnoid | UK Biobank | 111 | 89.4 | IVW | 1.07 | 0.92 | 1.24 | 0.36 | 104.9 | 0.62 |  |  | |  | |
| hemorrhage |  |  |  | WM | 1.05 | 0.81 | 1.35 | 0.72 |  |  |  |  | |  | |
|  |  |  |  | MR-Egger | 0.90 | 0.66 | 1.22 | 0.49 |  |  |  | 0.19 | | 94.6% | |
|  |  |  |  | MR-PRESSO | 1.07 | 0.93 | 1.24 | 0.35 |  |  |  |  | |  | |
| Intracerebral | UK Biobank | 111 | 89.4 | IVW | 1.16 | 0.99 | 1.36 | 0.07 | 97.1 | 0.81 |  |  | |  | |
| hemorrhage |  |  |  | WM | 1.21 | 0.94 | 1.56 | 0.14 |  |  |  |  | |  | |
|  |  |  |  | MR-Egger | 1.27 | 0.91 | 1.76 | 0.16 |  |  |  | 0.53 | | 94.7% | |
|  |  |  |  | MR-PRESSO | 1.16 | 0.99 | 1.35 | 0.06 |  |  |  |  | |  | |
| Subdural | UK Biobank | 111 | 89.4 | IVW | 1.41 | 1.08 | 1.83 | 0.01 | 96.2 | 0.82 |  |  | |  | |
| hemorrhage |  |  |  | WM | 1.32 | 0.87 | 2.00 | 0.19 |  |  |  |  | |  | |
|  |  |  |  | MR-Egger | 1.81 | 1.06 | 3.09 | 0.03 |  |  |  | 0.29 | | 94.7% | |
|  |  |  |  | MR-PRESSO | 1.41 | 1.10 | 1.80 | 0.01 |  |  |  |  | |  | |
| Arterial | UK Biobank | 110 | 89.3 | IVW | 1.32 | 1.13 | 1.53 | <0.001 | 127.8 | 0.11 |  |  | |  | |
| thrombo- |  |  |  | WM | 1.50 | 1.18 | 1.91 | 0.001 |  |  |  |  | |  | |
| embolism |  |  |  | MR-Egger | 1.34 | 0.98 | 1.83 | 0.06 |  |  |  | 0.89 | | 94.7% | |
|  |  |  |  | MR-PRESSO | 1.32 | 1.13 | 1.54 | 0.001 |  |  |  |  | |  | |
| Heart failure | HERMES | 110 | 89.3 | IVW | 1.26 | 1.21 | 1.30 | <0.001 | 233.0 | <0.001 |  |  | |  | |
|  |  |  |  | WM | 1.25 | 1.20 | 1.20 | <0.001 |  |  |  |  | |  | |
|  |  |  |  | MR-Egger | 1.21 | 1.12 | 1.30 | <0.001 |  |  |  | 0.26 | | 94.8% | |
|  |  |  |  | MR-PRESSO^a^ | 1.25 | 1.21 | 1.30 | <0.001 |  |  |  |  | |  | |

Appendix Table 1. (Continued)

| Outcomes | Sources | SNPs | *F*-statistic | Method | Odds | 95% CI |  | *P*-value | | | IVW | | |  | | MR-Egger | | | |  |
| --- | --- | --- | --- | --- | --- | --- | --- | --- | --- | --- | --- | --- | --- | --- | --- | --- | --- | --- | --- | --- |
|  |  |  |  |  | ratio |  |  |  | | | Cochran’s  *Q*-statistic | *P*-value | |  | | Intercept  *P*-value | | I^2^ | |  |
| T2DM | DIAMANTE | 110 | 89.3 | IVW | 1.03 | 1.00 | 1.07 | 0.09 | | | 365.1 | <0.001 | |  | |  | |  | |  |
|  |  |  |  | WM | 1.02 | 0.99 | 1.06 | 0.17 | | |  |  | |  | |  | |  | |  |
|  |  |  |  | MR-Egger | 1.00 | 0.94 | 1.08 | 0.91 | | |  |  | |  | | 0.36 | | 94.7% | |  |
|  |  |  |  | MR-PRESSO^a^ | 1.03 | 1.00 | 1.05 | 0.07 | | |  |  | |  | |  | |  | |  |
| Alzheimer’s | Jansen et al. | 110 | 89.3 | IVW | 1.00 | 1.00 | 1.01 | 0.37 | | | 134.3 | 0.05 | |  | |  | |  | |  |
| disease |  |  |  | WM | 1.00 | 0.99 | 1.01 | 0.51 | | |  |  | |  | |  | |  | |  |
|  |  |  |  | MR-Egger | 1.00 | 0.99 | 1.01 | 0.94 | | |  |  | |  | | 0.65 | | 96.6% | |  |
|  |  |  |  | MR-PRESSO | 1.00 | 1.00 | 1.01 | 0.37 | | |  |  | |  | |  | |  | |  |
|  |  |  |  |  | Mean | 95% CI |  | *P*-value | | |  |  | |  | |  | |  | |  |
|  |  |  |  |  | difference | |  | |  |  | | |  | |  | |  | |  | |
| Cognitive | Davies et al. | 109 | 89.9 | IVW | -0.001 | -0.007 | 0.004 | | 0.62 | 315.4 | | | <0.001 | |  | |  | |  | |
| function |  |  |  | WM | -0.006 | -0.013 | 0.000 | | 0.05 |  | | |  | |  | |  | |  | |
|  |  |  |  | MR-Egger | 0.001 | -0.011 | 0.013 | | 0.86 |  | | |  | |  | | 0.62 | | 94.7% | |
|  |  |  |  | MR-PRESSO^a^ | -0.002 | -0.008 | 0.002 | | 0.39 |  | | |  | |  | |  | |  | |

Abbreviations: CI, confidence interval; IVW, inverse variance weighting; MR, Mendelian randomization, SNP, single nucleotide polymorphism; WM, weighted median.

^a^MR-PRESSO estimate was obtained by excluding 3 outliers (*rs1458038, rs6689306, rs7574892*) for IHD 1000 Genomes; 2 outliers (*rs284277, rs3176326*) for any stroke and ischemic stroke, 2 outliers (rs11264280, rs4963776) for large artery stroke, and 1 outlier (rs284277) for small vessel stroke MEGASTROKE; 2 outliers (*rs10141892, rs3176326*) for heart failure HERMES; 6 outliers (*rs12245149, rs12604076, rs2540949, rs7650482, rs3176326, rs35005436*) for T2DM DIAMANTE, 3 outliers (*rs11191116, rs12245149, rs2885697*) for cognitive function using Davies et al.

Appendix Table 2. Association of genetically predicted ischemic heart disease (IHD), stroke, arterial thromboembolism, heart failure, type 2 diabetes (T2DM), cognitive function, and late-onset Alzheimer’s disease (AD) with atrial fibrillation using Mendelian randomization (MR)

| Exposures | Sources | SNPs | *F*-statistic | Method | Odds | 95% CI |  | *P*-value | IVW | |  | MR-Egger | |  |
| --- | --- | --- | --- | --- | --- | --- | --- | --- | --- | --- | --- | --- | --- | --- |
|  |  |  |  |  | ratio |  |  |  | Cochran’s  *Q*-statistic | *P*-value |  | Intercept  *P*-value | I^2^ | |
| IHD | 1000 Genomes | 39 | 61.9 | IVW | 1.12 | 1.06 | 1.18 | <0.001 | 138.2 | <0.001 |  |  |  | |
|  |  |  |  | WM | 1.14 | 1.08 | 1.19 | <0.001 |  |  |  |  |  | |
|  |  |  |  | MR-Egger | 1.14 | 1.00 | 1.29 | 0.05 |  |  |  | 0.77 | 91.9% | |
|  |  |  |  | MR-PRESSO^a^ | 1.12 | 1.08 | 1.17 | <0.001 |  |  |  |  |  | |
| Any stroke^b^ | MEGASTROKE | 7 | 39.7 | IVW | 1.18 | 1.04 | 1.35 | 0.01 | 14.3 | 0.03 |  |  |  | |
|  |  |  |  | WM | 1.13 | 0.99 | 1.28 | 0.07 |  |  |  |  |  | |
|  |  |  |  | MR-Egger | 0.54 | 0.21 | 1.36 | 0.19 |  |  |  | 0.10 | 0.0% | |
|  |  |  |  | MR-PRESSO^a^ | 1.18 | 1.00 | 1.39 | 0.05 |  |  |  |  |  | |
| Ischemic stroke^b^ | MEGASTROKE | 9 | 37.7 | IVW | 1.17 | 1.04 | 1.31 | 0.01 | 20.2 | 0.01 |  |  |  | |
|  |  |  |  | WM | 1.10 | 0.98 | 1.24 | 0.09 |  |  |  |  |  | |
|  |  |  |  | MR-Egger | 0.85 | 0.28 | 2.62 | 0.78 |  |  |  | 0.58 | 27.4% | |
|  |  |  |  | MR-PRESSO^a^ | 1.12 | 1.00 | 1.26 | 0.06 |  |  |  |  |  | |
| Large artery | MEGASTROKE | 4 | 37.3 | IVW | 1.03 | 0.95 | 1.11 | 0.48 | 8.4 | 0.04 |  |  |  | |
| stroke |  |  |  | WM | 1.02 | 0.97 | 1.08 | 0.45 |  |  |  |  |  | |
|  |  |  |  | MR-Egger | 0.90 | 0.76 | 1.06 | 0.19 |  |  |  | 0.08 | 0.0% | |
|  |  |  |  | MR-PRESSO^a^ | 1.03 | 0.91 | 1.16 | 0.53 |  |  |  |  |  | |
| Cardioembolic | MEGASTROKE | 4 | 73.7 | IVW | 2.67 | 1.59 | 4.49 | <0.001 | 509.8 | <0.001 |  |  |  | |
| stroke |  |  |  | WM | 3.09 | 2.61 | 3.65 | <0.001 |  |  |  |  |  | |
|  |  |  |  | MR-Egger | 1.65 | 0.54 | 5.00 | 0.38 |  |  |  | 0.33 | 90.4% | |
|  |  |  |  | MR-PRESSO^a^ | 2.67 | 1.15 | 6.20 | 0.03 |  |  |  |  |  | |
| Small vessel | MEGASTROKE | 33 | 23.4 | IVW | 1.02 | 1.00 | 1.04 | 0.06 | 35.9 | 0.29 |  |  |  | |
| stroke |  |  |  | WM | 1.03 | 0.99 | 1.06 | 0.11 |  |  |  |  |  | |
|  |  |  |  | MR-Egger | 1.04 | 0.99 | 1.09 | 0.13 |  |  |  | 0.48 | 0.0% | |
|  |  |  |  | MR-PRESSO^a^ | 1.02 | 1.00 | 1.04 | 0.07 |  |  |  |  |  | |

Appendix Table 2. (Continued)

| Exposures | Sources | | SNPs | | *F*-statistic | | Method | | Odds | | 95% CI | | |  | | *P*-value | IVW | | |  | MR-Egger | | |
| --- | --- | --- | --- | --- | --- | --- | --- | --- | --- | --- | --- | --- | --- | --- | --- | --- | --- | --- | --- | --- | --- | --- | --- |
|  |  | |  | |  | |  | | ratio | | | |  |  | |  | Cochran’s  *Q*-statistic | | *P*-value |  | Intercept  *P*-value | | I^2^ |
| Hemorrhagic | | UK Biobank | | 9 | | 23.1 | | IVW | | 1.02 | | 0.98 | | | 1.05 | 0.33 | 11.2 | 0.19 | |  |  |  | |
| stroke | |  | |  | |  | | WM | | 0.99 | | 0.95 | | | 1.04 | 0.77 |  |  | |  |  |  | |
|  | |  | |  | |  | | MR-Egger | | 0.97 | | 0.90 | | | 1.06 | 0.53 |  |  | |  | 0.27 | 0.0% | |
|  | |  | |  | |  | | MR-PRESSO | | 1.02 | | 0.98 | | | 1.06 | 0.36 |  |  | |  |  |  | |
| Subarachnoid | | UK Biobank | | 13 | | 22.1 | | IVW | | 0.99 | | 0.98 | | | 1.01 | 0.52 | 16.3 | 0.18 | |  |  |  | |
| hemorrhage | |  | |  | |  | | WM | | 1.00 | | 0.98 | | | 1.03 | 0.73 |  |  | |  |  |  | |
|  | |  | |  | |  | | MR-Egger | | 1.02 | | 0.99 | | | 1.06 | 0.25 |  |  | |  | 0.08 | 0.0% | |
|  | |  | |  | |  | | MR-PRESSO | | 0.99 | | 0.97 | | | 1.01 | 0.53 |  |  | |  |  |  | |
| Intracerebral | | UK Biobank | | 6 | | 22.9 | | IVW | | 1.02 | | 0.98 | | | 1.05 | 0.31 | 11.6 | 0.04 | |  |  |  | |
| hemorrhage | |  | |  | |  | | WM | | 1.00 | | 0.97 | | | 1.03 | 0.95 |  |  | |  |  |  | |
|  | |  | |  | |  | | MR-Egger | | 0.88 | | 0.78 | | | 0.99 | 0.03 |  |  | |  | 0.02 | 0.0% | |
|  | |  | |  | |  | | MR-PRESSO | | 1.02 | | 0.98 | | | 1.06 | 0.36 |  |  | |  |  |  | |
| Subdural | | UK Biobank | | 5 | | 23.0 | | IVW | | 1.00 | | 1.00 | | | 1.00 | 0.38 | 2.5 | 0.64 | |  |  |  | |
| hemorrhage | |  | |  | |  | | WM | | 1.00 | | 1.00 | | | 1.00 | 0.46 |  |  | |  |  |  | |
|  | |  | |  | |  | | MR-Egger | | 1.00 | | 1.00 | | | 1.00 | 0.39 |  |  | |  | 1.00 | 81.2% | |
|  | |  | |  | |  | | MR-PRESSO | | 1.00 | | 1.00 | | | 1.00 | 0.33 |  |  | |  |  |  | |
| Arterial | | UK Biobank | | 5 | | 23.2 | | IVW | | 1.02 | | 0.98 | | | 1.07 | 0.32 | 9.9 | 0.04 | |  |  |  | |
| thrombo- | |  | |  | |  | | WM | | 1.03 | | 0.99 | | | 1.07 | 0.18 |  |  | |  |  |  | |
| embolism | |  | |  | |  | | MR-Egger | | 1.05 | | 0.94 | | | 1.07 | 0.40 |  |  | |  | 0.62 | 0.0% | |
|  | |  | |  | |  | | MR-PRESSO^a^ | | 1.04 | | 1.00 | | | 1.08 | 0.06 |  |  | |  |  |  | |
| Heart failure | | HERMES | | 9 | | 35.7 | | IVW | | 1.86 | | 1.30 | | | 2.66 | 0.001 | 111.2 | <0.001 | |  |  |  | |
|  | |  | |  | |  | | WM | | 1.68 | | 1.40 | | | 2.01 | <0.001 |  |  | |  |  |  | |
|  | |  | |  | |  | | MR-Egger | | 1.78 | | 0.46 | | | 6.92 | 0.41 |  |  | |  | 0.95 | 0.0% | |
|  | |  | |  | |  | | MR-PRESSO | | 1.60 | | 1.39 | | | 1.85 | 0.001 |  |  | |  |  |  | |

Appendix Table 2. (Continued)

| Exposures | Sources | SNPs | *F*-statistic | Method | Odds | 95% CI | |  | *P*-value | IVW | | | |  | | MR-Egger | | | | |  |
| --- | --- | --- | --- | --- | --- | --- | --- | --- | --- | --- | --- | --- | --- | --- | --- | --- | --- | --- | --- | --- | --- |
|  |  |  |  |  | ratio | |  |  |  | Cochran’s  *Q*-statistic | | *P*-value | |  | | Intercept  *P*-value | | I^2^ | | |  |
| T2DM | DIAMANTE | 195 | 78.1 | IVW | 1.00 | 0.97 | | 1.03 | 0.92 | 503.9 | <0.001 | |  | |  | |  | | |  |  |
|  |  |  |  | WM | 0.97 | 0.94 | | 1.00 | 0.06 |  |  | |  | |  | |  | | |  |  |
|  |  |  |  | MR-Egger | 0.95 | 0.90 | | 1.01 | 0.10 |  |  | |  | | 0.06 | | 93.4% | | |  |  |
|  |  |  |  | MR-PRESSO^a^ | 1.00 | 0.97 | | 1.02 | 0.69 |  |  | |  | |  | |  | | |  |  |
| Cognitive | Davies et al. | 103 | 41.2 | IVW | 0.80 | 0.60 | | 1.07 | 0.13 | 222.3 | | <0.001 | |  | |  | | |  | | |
| function |  |  |  | WM | 0.67 | 0.50 | | 0.89 | 0.01 |  | |  | |  | |  | | |  | | |
|  |  |  |  | MR-Egger | 1.17 | 0.33 | | 4.14 | 0.81 |  | |  | |  | | 0.56 | | | 2.3% | | |
|  |  |  |  | MR-PRESSO^a^ | 0.75 | 0.59 | | 0.96 | 0.02 |  | |  | |  | |  | | |  | | |
| Alzheimer’s | Jansen et al. | 27 | 163.6 | IVW | 0.94 | 0.85 | | 1.03 | 0.20 | 29.4 | | 0.29 | |  | |  | | |  | | |
| disease |  |  |  | WM | 0.97 | 0.87 | | 1.09 | 0.60 |  | |  | |  | |  | | |  | | |
|  |  |  |  | MR-Egger | 1.00 | 0.88 | | 1.13 | 0.94 |  | |  | |  | | 0.15 | | | 98.8% | | |
|  |  |  |  | MR-PRESSO | 0.94 | 0.85 | | 1.04 | 0.21 |  | |  | |  | |  | | |  | | |

Abbreviations: CI, confidence interval; IVW, inverse variance weighting; MR, Mendelian randomization, SNP, single nucleotide polymorphism; WM, weighted median.

^a^MR-PRESSO estimate was obtained by excluding 3 outliers (*rs11191416, rs12202017, rs6689306*) for IHD 1000 Genomes; 1 outlier (*rs635634)* for ischemic stroke MEGASTROKE; 1 outlier (*rs10105568)* for arterial thromboembolism UK Biobank; 3 outliers (*rs11745324, rs17617337, rs4746140*) for heart failure HERMES; 7 outliers (*rs12811407, rs1421085, rs17836088, rs55653563, rs62107261, rs7608050, rs76895963*) for T2DM DIAMANTE; 2 outliers (*rs10761765, rs242559*) for cognitive function using Davies et al.

^b^MR estimates for any and ischemic stroke MEGASTROKE excluded 1 SNP (*rs2634074*) as well as heart failure HERMES excluded 1 SNP (*rs17042102*) because of extremely high heterogeneity of MR estimate from that SNP based on MR-Egger results.

Appendix Table 3. Sensitivity analyses for association of genetically predicted atrial fibrillation with cognitive function and genetically predicted cardioembolic stroke with atrial fibrillation using Mendelian randomization (MR) after excluding overlapping SNPs^a^

| Exposures | Outcomes | SNPs | *F*-statistic | Method | Mean | 95% CI |  | *P*-value | IVW | | | |  | | MR-Egger | | | |
| --- | --- | --- | --- | --- | --- | --- | --- | --- | --- | --- | --- | --- | --- | --- | --- | --- | --- | --- |
|  |  |  |  |  | difference |  |  |  | Cochran’s  *Q*-statistic | | *P*-value | |  | | Intercept  *P*-value | | I^2^ | |
| Atrial | Cognitive | 108 | 90.4 | IVW | -0.002 | -0.008 | 0.003 | 0.41 | 260.8 | <0.001 | |  | |  | |  | |  |
| fibrillation | function |  |  | WM | -0.006 | -0.013 | 0.000 | 0.06 |  |  | |  | |  | |  | |  |
|  |  |  |  | MR-Egger | 0.003 | -0.008 | 0.014 | 0.58 |  |  | |  | | 0.27 | | 94.7% | |  |
|  |  |  |  | MR-PRESSO^b^ | -0.003 | -0.008 | 0.002 | 0.20 |  |  | |  | |  | |  | |  |
|  |  |  |  |  |  |  |  |  |  |  | |  | |  | |  | |  |
| Exposures | Outcomes | SNPs | *F*-statistic | Method | Odds ratio | 95% CI |  | *P*-value | IVW | | | |  | | MR-Egger | | | |
|  |  |  |  |  |  |  |  |  | Cochran’s  *Q*-statistic | | *P*-value | |  | | Intercept  *P*-value | | I^2^ | |
| Cardioembolic | Atrial | 3 | 81.1 | IVW | 2.68 | 1.33 | 5.40 | 0.006 | 509.5 | <0.001 | |  | |  | |  | |  |
| stroke | fibrillation |  |  | WM | 3.02 | 2.48 | 3.66 | <0.001 |  |  | |  | |  | |  | |  |
|  |  |  |  | MR-Egger | 1.61 | 0.35 | 7.34 | 0.54 |  |  | |  | | 0.44 | | 93.1% | |  |
|  |  |  |  | MR-PRESSO | NA |  |  |  |  |  | |  | |  | |  | |  |
| Heart failure | Atrial | 8 | 35.5 | IVW | 1.62 | 1.23 | 2.14 | 0.001 | 54.9 | <0.001 | |  | |  | |  | |  |
|  | fibrillation |  |  | WM | 1.67 | 1.42 | 1.97 | <0.001 |  |  | |  | |  | |  | |  |
|  |  |  |  | MR-Egger | 1.41 | 0.51 | 3.86 | 0.51 |  |  | |  | | 0.78 | | 0.0% | |  |
|  |  |  |  | MR-PRESSO | 1.60 | 0.06 | 1.38 | 0.001 |  |  | |  | |  | |  | |  |

Abbreviations: CI, confidence interval; IVW, inverse variance weighting; MR, Mendelian randomization, NA, not applicable, SNP, single nucleotide polymorphism; WM, weighted median.

^a^Association of atrial fibrillation with cognitive function was obtained after excluding 1 SNP (*rs11191116*), association of cardioembolic stroke with atrial fibrillation was obtained after excluding 1 SNP (*rs12932445*), and association of heart failure with atrial fibrillation was obtained after excluding 1 SNP (*rs4746140*).

^b^MR-PRESSO estimate was obtained by excluding 3 outliers (*rs11598047, rs12245149, rs2885697*) for cognitive function using Davies et al.

Appendix Table 4. Power calculations for bi-directional associations of genetically predicted atrial fibrillation with various diseases

| Exposures | Outcomes | SNPs | R^2^ | Minimum effect size with 80% power to detect |
| --- | --- | --- | --- | --- |
| Atrial fibrillation | Ischemic heart disease | 108 | 0.00934 | 1.15 |
| Atrial fibrillation | Any stroke | 109 | 0.00940 | 1.15 |
| Atrial fibrillation | Ischemic stroke | 109 | 0.00940 | 1.16 |
| Atrial fibrillation | Large artery stroke | 110 | 0.00948 | 1.44 |
| Atrial fibrillation | Cardioembolic stroke | 110 | 0.00948 | 1.34 |
| Atrial fibrillation | Small vessel stroke | 110 | 0.00948 | 1.39 |
| Atrial fibrillation | Hemorrhagic stroke | 110 | 0.00944 | 1.68 |
| Atrial fibrillation | Subarachnoid hemorrhage | 111 | 0.00954 | 2.01 |
| Atrial fibrillation | Intracerebral hemorrhage | 111 | 0.00954 | 2.08 |
| Atrial fibrillation | Subdural hemorrhage | 111 | 0.00954 | 2.77 |
| Atrial fibrillation | Arterial thromboembolism | 110 | 0.00944 | 1.95 |
| Atrial fibrillation | Heart failure | 110 | 0.00944 | 1.14 |
| Atrial fibrillation | Type 2 diabetes | 110 | 0.00944 | 1.11 |
| Atrial fibrillation | Alzheimer’s disease | 110 | 0.00944 | 1.10 |
| Atrial fibrillation | Cognitive function | 109 | 0.00942 | 0.05 |
|  |  |  |  |  |
| Ischemic heart disease | Atrial fibrillation | 39 | 0.0129 | 1.10 |
| Any stroke | Atrial fibrillation | 7 | 0.0006 | 1.48 |
| Ischemic stroke | Atrial fibrillation | 9 | 0.0008 | 1.43 |
| Large artery stroke | Atrial fibrillation | 4 | 0.0004 | 1.63 |
| Cardioembolic stroke | Atrial fibrillation | 4 | 0.0007 | 1.45 |
| Small vessel stroke | Atrial fibrillation | 33 | 0.0019 | 1.27 |
| Hemorrhagic stroke | Atrial fibrillation | 9 | 0.0005 | 1.52 |
| Subarachnoid hemorrhage | Atrial fibrillation | 13 | 0.0007 | 1.45 |
| Intracerebral hemorrhage | Atrial fibrillation | 6 | 0.0003 | 1.65 |
| Subdural hemorrhage | Atrial fibrillation | 5 | 0.0003 | 1.71 |
| Arterial thromboembolism | Atrial fibrillation | 5 | 0.0003 | 1.71 |
| Heart failure | Atrial fibrillation | 9 | 0.0003 | 1.66 |
| Type 2 diabetes | Atrial fibrillation | 195 | 0.0167 | 1.09 |
| Alzheimer’s disease | Atrial fibrillation | 103 | 0.0092 | 1.12 |
| Cognitive function | Atrial fibrillation | 27 | 0.0145 | 1.10 |

Abbreviations: SNP, single nucleotide polymorphism; R^2^, variance explained

**Any stroke Ischemic stroke Cardioembolic stroke Hemorrhagic stroke**


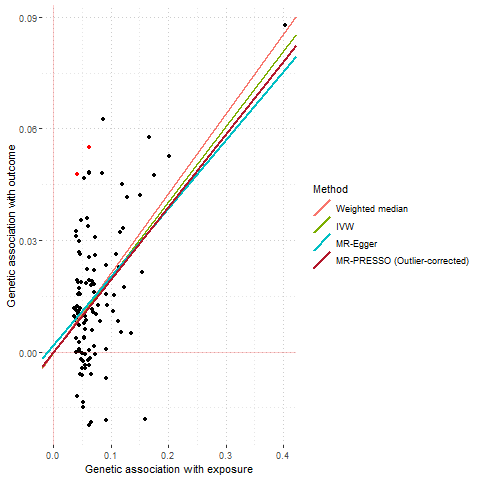

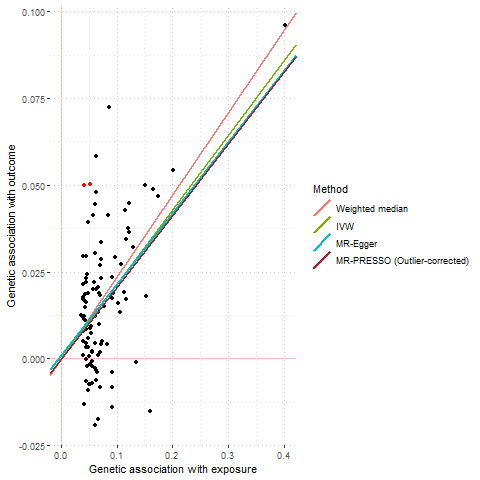

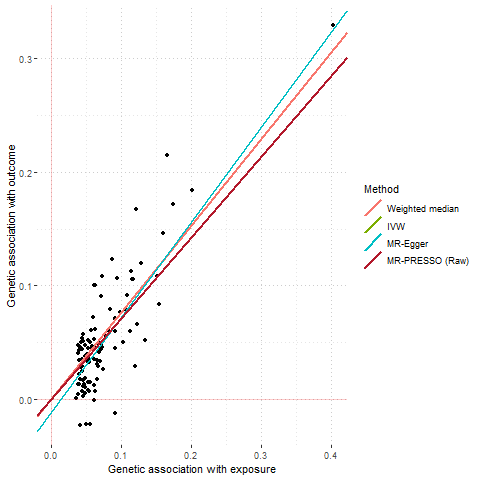

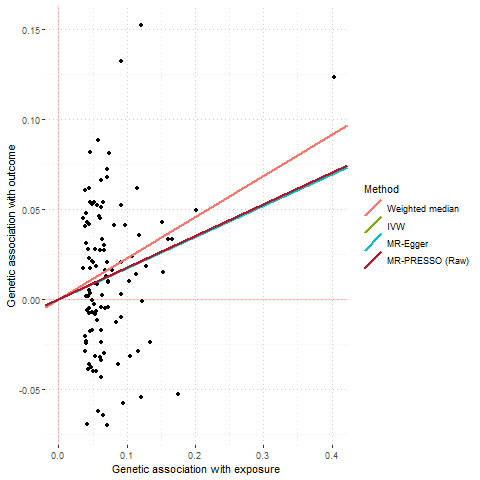


**Intracerebral hemorrhage Subdural hemorrhage Arterial thromboembolism Heart failure**


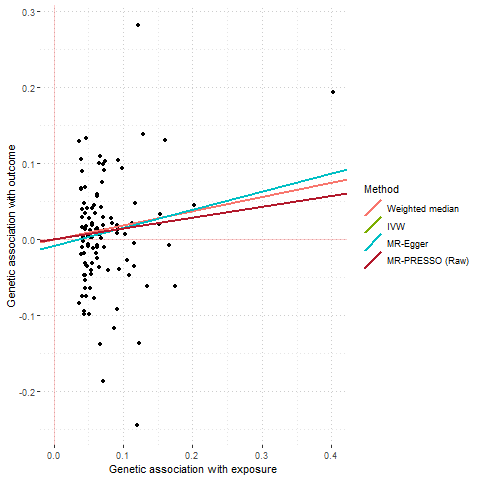

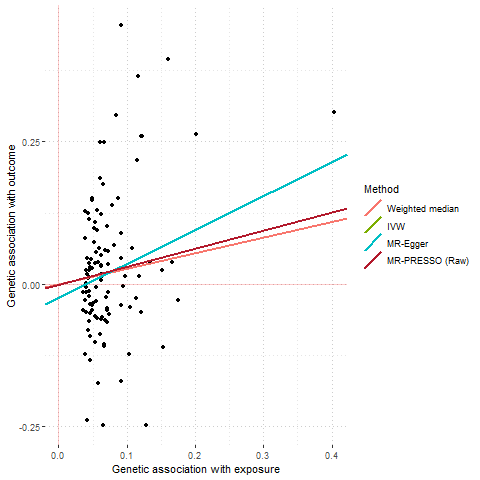

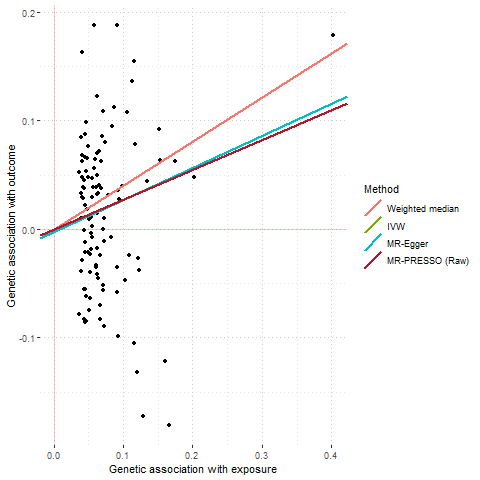

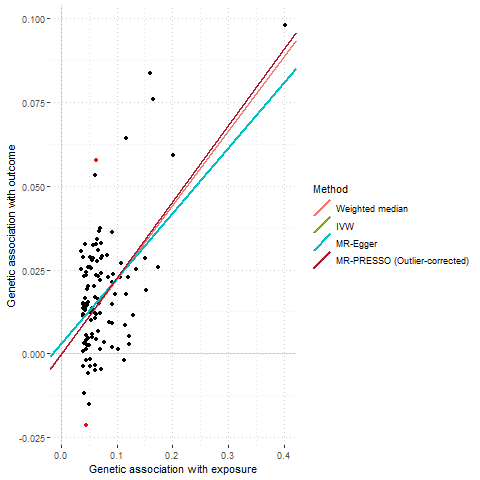


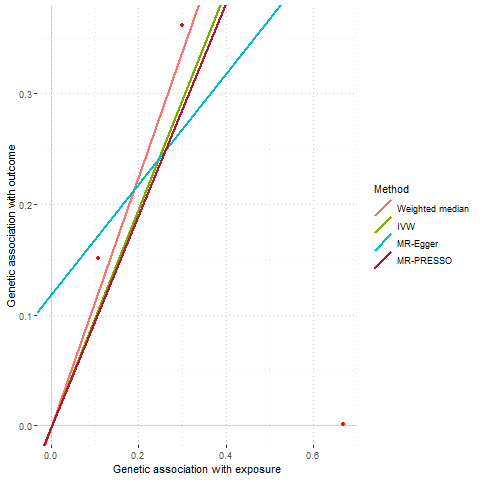

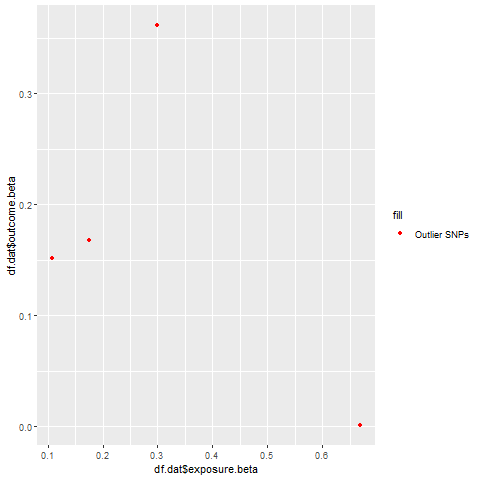


Appendix Figure 1. Scatter plots for associations of atrial fibrillation with cardiovascular diseases reaching at least suggestive significance (*P*<0.1)

**Ischemic heart disease Any stroke Ischemic stroke Cardioembolic stroke**


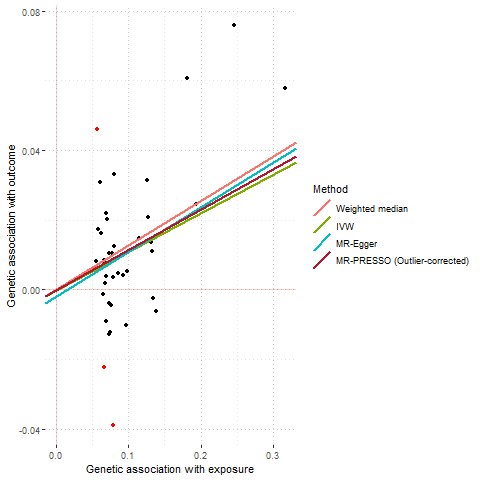

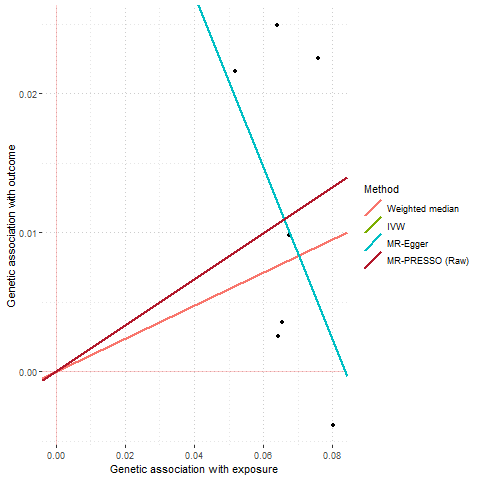

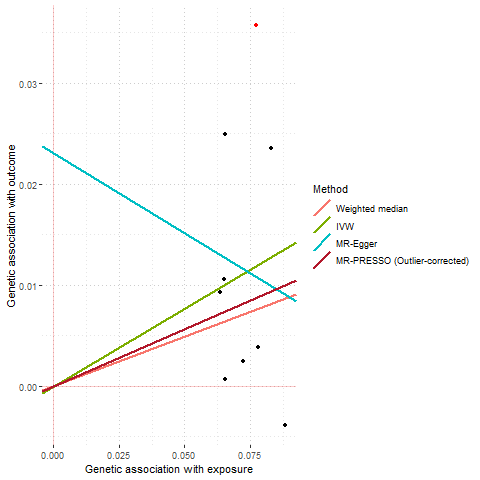

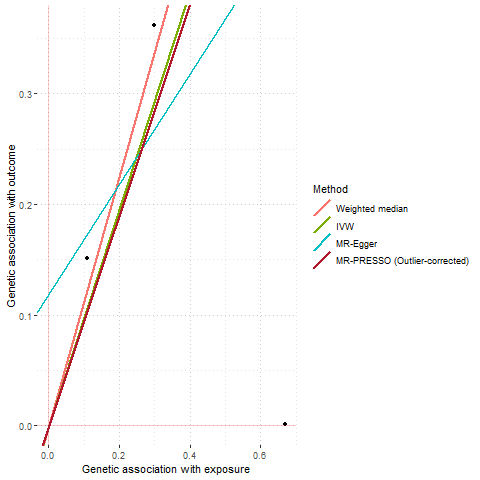


**Small vessel stroke Heart failure**


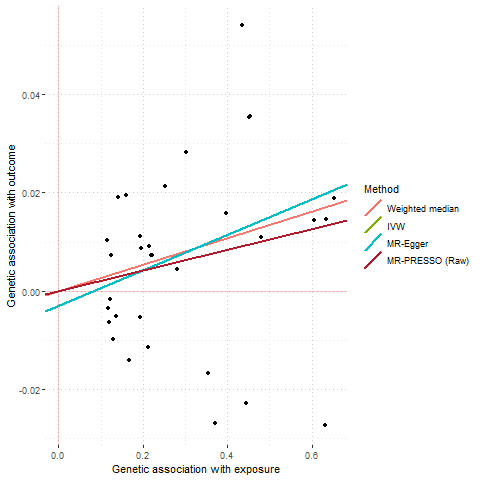

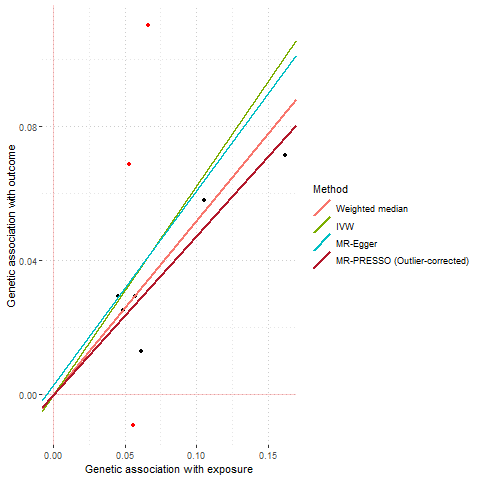


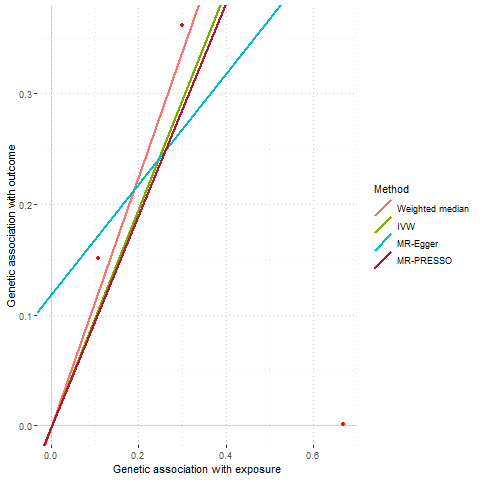

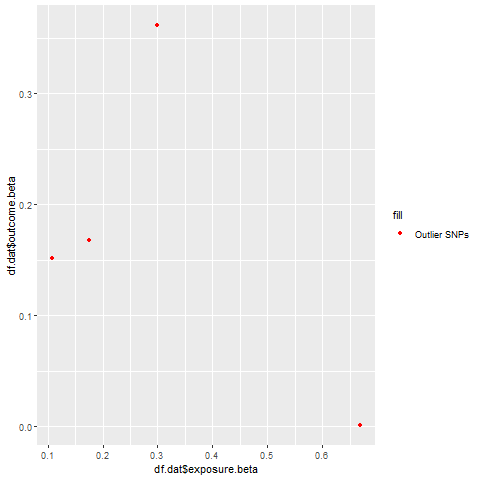


Appendix Figure 2. Scatter plots for associations of cardiovascular diseases with atrial fibrillation reaching at least suggestive significance (*P*<0.1)
